# Supplementary figures and images for: Psychobiotics in Depression: Sources, Metabolites, and Treatment—A Systematic Review
Source: Nutrients. 2025 Jun 27;17(13):2139. doi: 10.3390/nu17132139 (PMC12252283; doi:10.3390/nu17132139)

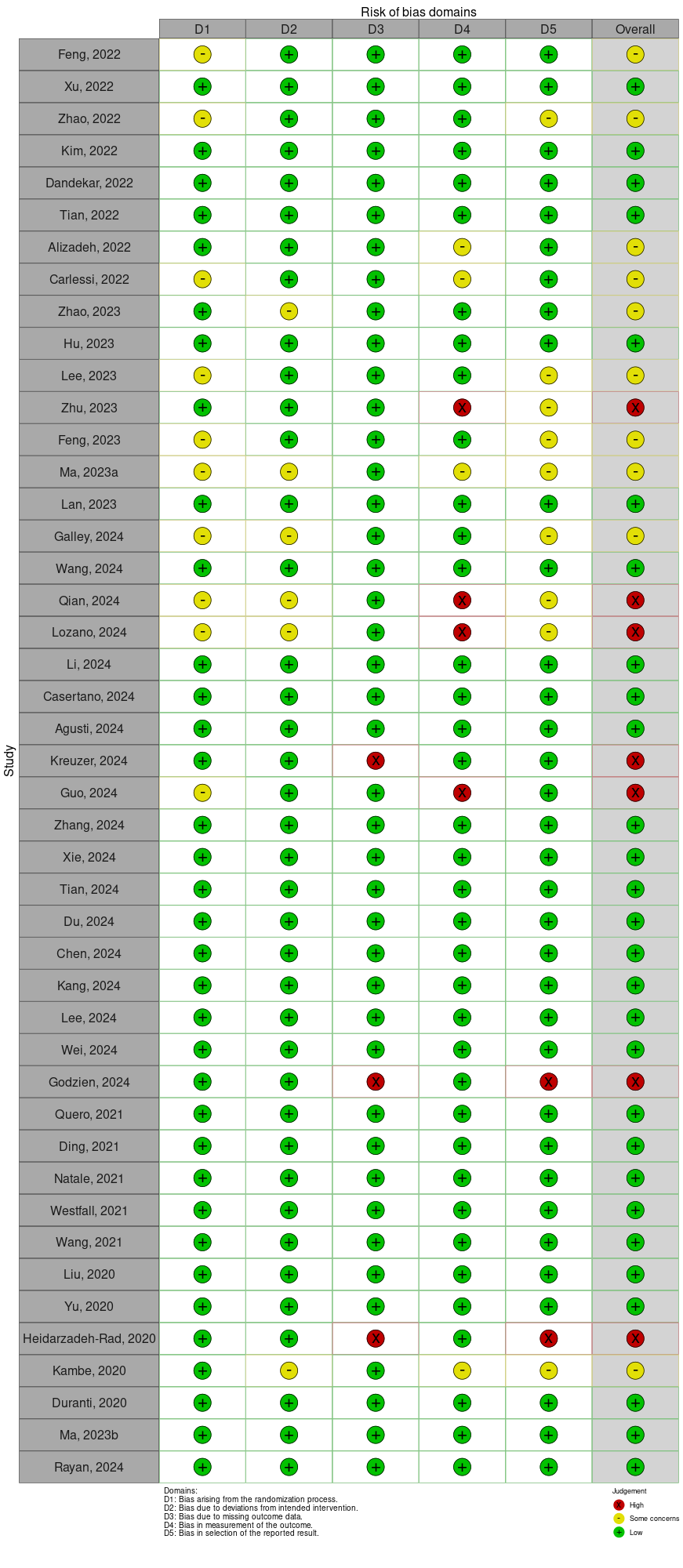

Supplement: Supplementary file 1 [file nutrients-17-02139-s001.zip › Fig. S2.tif]

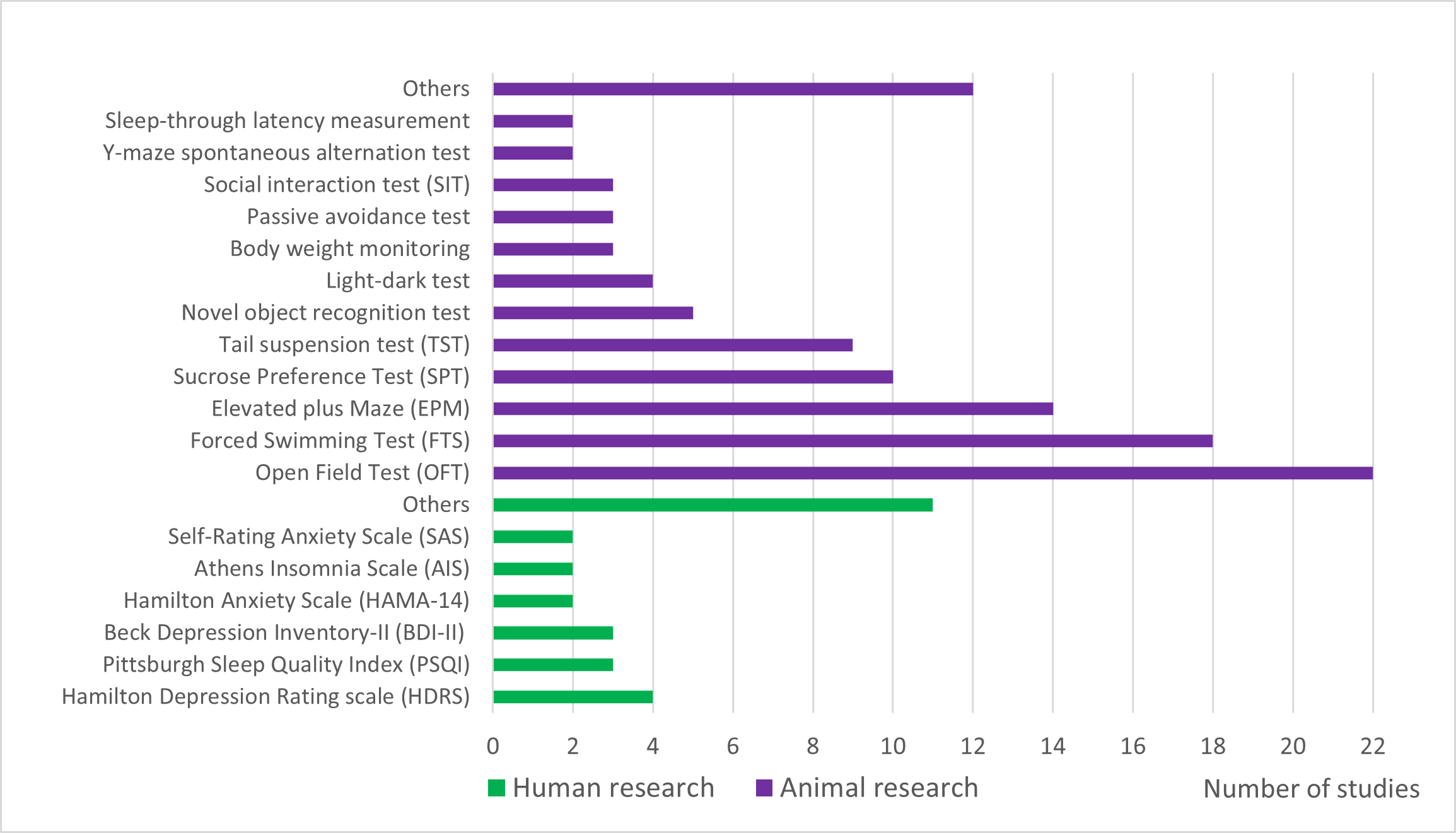

Supplement: Supplementary file 1 [file nutrients-17-02139-s001.zip › Fig. S1.tif]
